# Intrabody Targeting HIF-1α Mediates Transcriptional Downregulation of Target Genes Related to Solid Tumors

**DOI:** 10.3390/ijms222212335

**Published:** 2021-11-15

**Authors:** Yaozhong Hu, Ema Romão, Cécile Vincke, Lea Brys, Yvon Elkrim, Marylène Vandevenne, Changxiao Liu, Serge Muyldermans

**Affiliations:** 1 Research Institute of Public Health, School of Medicine, Nankai University, Tianjin 300071, China; yzhu@nankai.edu.cn; 2 Laboratory of Cellular and Molecular Immunology, Vrije Universiteit Brussel, Pleinlaan 2, 1050 Brussels, Belgium; Ema.Estevens.Romao@vub.be (E.R.); Cecile.Vincke@vub.be (C.V.); Lea.Brys@vub.ac.be (L.B.); Yvon.Elkrim@vub.ac.be (Y.E.); 3 Chargé de Recherche FRS-FNRS, Macromolécules Biologiques, Centre D’ingénierie des Proteins, Université de Liège, 4000 Liège, Belgium; mvandevenne@ulg.ac.be; 4 State Key Laboratory of Drug Delivery Technology and Pharmacokinetics, Tianjin Institute of Pharmaceutical Research, Tianjin 300301, China

**Keywords:** HIF-1α, nanobodies, transcriptional activation, target genes

## Abstract

Uncontrolled growth of solid tumors will result in a hallmark hypoxic condition, whereby the key transcriptional regulator of hypoxia inducible factor-1α (HIF-1α) will be stabilized to activate the transcription of target genes that are responsible for the metabolism, proliferation, and metastasis of tumor cells. Targeting and inhibiting the transcriptional activity of HIF-1 may provide an interesting strategy for cancer therapy. In the present study, an immune library and a synthetic library were constructed for the phage display selection of Nbs against recombinant PAS B domain protein (rPasB) of HIF-1α. After panning and screening, seven different nanobodies (Nbs) were selected, of which five were confirmed via immunoprecipitation to target the native HIF-1α subunit. The inhibitory effect of the selected Nbs on HIF-1 induced activation of target genes has been evaluated after intracellular expression of these Nbs in HeLa cells. The dramatic inhibition of both intrabody formats on the expression of HIF-1-related target genes has been confirmed, which indicated the inhibitory efficacy of selected Nbs on the transcriptional activity of HIF-1.

## 1. Introduction

An appropriate level of oxygen supply is critical for the regular energy metabolism and signaling transmission in healthy cells [1,2]. The uncontrolled growth of solid tumors will consume a lot of oxygen, and install a hypoxic condition [3,4]. Meanwhile, the rapidly growing tumor mass will cause damage to the vascular system resulting in a disrupted oxygen supply [5]. However, the flexible adaptive changes of tumor cells allow them to avoid hypoxia-induced cell death. The activation of particular genes and signaling cascades permits tumor cells to survive hypoxia [6,7]. Furthermore, such genetic adaptation can make tumors more aggressive and resistant to conventional radiotherapy and chemotherapy [8,9], leading to a poor clinical prognosis [10,11]. Under hypoxic condition, tumor cells can convert energy metabolism patterns and transcriptionally activate related genes for the adaptation and response of tumor cells to hypoxia [12].

Hypoxia inducible factor-1 (HIF-1) serves as the key regulator responsible for the transcriptional activation of target genes in tumors [13,14]. HIF-1 is a heterodimer of HIF-1α and HIF-1β subunits, both of which belong to the basic helix–loop–helix/Per-ARNT-SIM (bHLH-PAS) protein family. HIF-1β subunit is constitutively expressed in mammalian cells, whereas the level of the HIF-1αis oxygen-dependent and regulated by a sophisticated ubiquitin-proteasome degradation system [15]. Under normoxic condition (oxygen tension around 21%), cytoplasmic HIF-1α is hydroxylated by a family of prolyl hydroxylase domains (PHDs) on proline residues 402 and 564 located within the oxygen-dependent degradation domain (ODDD) [16]. The hydroxylation of these prolines is the key mechanism of negative regulation of HIF-1α activity and results in the formation of the von Hippel–Lindau protein (pVHL) E3 ligase complex [17], which ubiquitinates HIF-1α, resulting in proteasomal degradation of this subunit [18]. Under hypoxic condition (oxygen tension lower than 5%), HIF-1α forms a complex with the β subunit, resulting in the formation of a fully functional HIF-1 transcriptional factor [19]. The heterodimerization will result in the successful translocation of HIF-1 to the nucleus, where it will associate with hypoxia-inducible response elements (HREs) in the promoters of target genes [20]. Then, the transcription factor can activate target genes related to metabolism, growth, and proliferation processes in tumor cells, to overcome the inhibitions from a low oxygen level [21,22].

The PAS domain of HIF-1α plays a key role during the heterodimerization and is responsible for the interaction between α and β subunits [23,24]. The deletion of PAS B domain dramatically decreased the level of HIF-1 in cells, and even a few amino acid mutations in the PAS B domain can result in a lower transcriptional activity of HIF-1 [25]. Herein, the targeting to PAS B domain of HIF-1α to interfere with the heterodimerization of two subunits may provide an attractive therapeutic strategy. Whereas cancer therapy with conventional antibodies has been very successful in targeting the extracellular tumor markers [26,27,28], no notable results have been obtained against tumor markers residing in the cytoplasm or nucleus. A steadily growing interest is going into the application of antibody fragments, especially nanobodies (Nbs) derived from the naturally occurring heavy chain-only antibodies (HCAbs) in camelids [29]. Such Nbs are the smallest antibody fragments with full retention of antigen binding capacity and specificity of the original IgG. The molecular weight of Nbs is around 1/10 of that of conventional antibodies [30]. Because of their small size, and a concomitant expected lower immunogenicity risk [31], Nbs are an appropriate therapeutic tool and because of their robustness they might also be considered as the best possible antibody-derived candidate for intracellular targeting of tumor markers in the cytoplasm or the nucleus. It has been reported that Nbs expressed in mammalian cells (referred to as intrabodies) retain their full antigen binding capacity [32]. In previous reports, intrabodies have been developed to target PHD2 [33] or ODDD [34] to result in the stabilization or oxygen-induced degradation of HIF-1α. In this study, Nbs were employed as the candidate to target the PAS B domain to inhibit the heterodimerization of HIF-1α and β subunits and, as a result, to inhibit the transcription of HIF-1 related target genes.

In the present study, several Nbs against the PAS B domain of HIF-1α have been retrieved from immune and synthetic Nb libraries. The targeting of selected Nbs to endogenous HIF-1α has been demonstrated. Selected Nbs have been produced in tumor cells and their inhibitory effects were determined by quantifying the level of HIF-1 target genes. These intrabodies provide a potential strategy for the specific interaction with endogenous proteins or tumor progression related targets in mammalian cells.

## 2. Results

### 2.1. Preparation of rPasB Proteins

Recombinant PAS B domain protein (rPasB) protein was produced with a hexahistidine tag (His-tag) at its C-terminal end for purification and detection. rPasB was expressed in BL21(DE3) after induction with isopropyl-β-D-thiogalactopyranoside (IPTG), released from the cytoplasm and purified by immobilized metal affinity chromatography (IMAC) and size exclusion chromatography (SEC). The purity and molecular mass of rPasB protein were determined by sodium dodecyl sulfate-polyacrylamide gel electrophoresis (SDS-PAGE) under reducing and non-reducing conditions, stained with Coomassie. Western blot was performed to verify the presence of the His-tag. The identification of rPasB has been depicted in the previous publication [35], and the data were included as Figure S1, which indicated that a single band at the position slightly below the 15 kDa was visualized on the SDS-PAGE gel to indicate the acceptable purity of rPasB. Western blot under reducing and non-reducing conditions revealed the presence of the His-tag. In order to assess the stability of rPasB protein, the melting temperature (Tm) of rPasB has been determined by circular dichroism (CD), which indicated a Tm of around 44.2 °C after non-linear fitting (Figure S2).

### 2.2. Construction of the Libraries, Panning, and Screening

After immunization of a dromedary with rPasB protein, the immune library was constructed in pMECS. The size of the Nb library was determined to comprise about 3.3 × 10^8^ individual transformants in *Escherichia coli* (*E. coli*) TG1 cells. A colony PCR on 95 randomly chosen individual colonies was performed with primers MP57 and GIII, which will anneal on the upstream and downstream regions of the cloned Nb gene fragments in the phagemid, and the PCR amplicons were separated by 1% agarose gel electrophoresis. An amplicon with the size of around 600 bp is expected for clones carrying the Nb gene. From the result of the agarose gel electrophoresis (73 out of 95 clones yielded an amplicon of around 600 bp), it was estimated that about 77% of the colonies within the library comprise cells containing phagemids possessing an insert with the correct size for a Nb gene.

**Figure 1:**
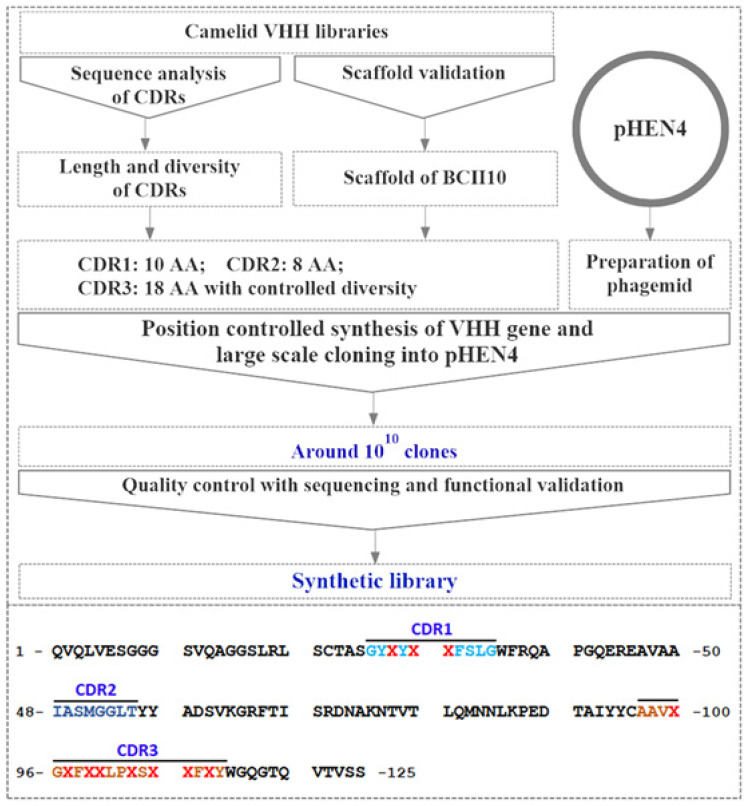
Schematic illustration of the strategy used to construct the synthetic library. The selected scaffold of the Nb is BCII10. A fixed size of 10 aa was chosen for the CDR1, 8 aa for CDR2, and 18 aa for CDR3 with controlled diversity. Finally, the pHEN4 phagemid was chosen to construct the library. The synthetic randomized genes were cloned into the pHEN4 vector, and then transformed into TG1 cells. The diversity of the synthetic library was controlled by the PCR analysis and sequencing. For the amino acid sequence of the CDR regions, the underlined residues have not been changed. Residues with strikethrough indicated the change and will introduce the diversity for the synthetic library.

For the construction of the synthetic library, VHH genes were designed in which codons of complementarity determining regions (CDRs) were randomized with a bias for Ser, Tyr, Arg, Lys, Gly, and Asn (Figure 1). About 9.63 × 10^10^ individual transformants were obtained for the synthetic library [36]. To ensure the quality of the library, a colony PCR was performed on 100 randomly selected clones, and the result after agarose gel electrophoresis revealed the presence of correctly sized insert for a VHH gene in the phagemids within 80% of the clones. The diversity and representation of randomized codons within the library was then confirmed by sequencing 100 inserts.

For bio-panning and screening against rPasB protein, a representative fraction of the bacterial library of the cloned Nb repertoire (1 × 10^10^ TG1 cells from the immune library stock and 1.6 × 10^11^ cells from the synthetic library stock) was cultured and infected with VCSM13 helper phages to express the Nb at the tip of M13 virion particles. The immune library and synthetic library were used separately for bio-panning on immobilized rPasB. Enrichment was determined by comparing the colony numbers from the wells with or without coated antigen. An increasing ratio of eluted phages was observed from 1.1 to 42 during the consecutive rounds of bio-panning of the immune library, and from 1 to 25 for the synthetic library.

For screening of clones from the immune library, 190 individual colonies were selected from the 2nd (32 colonies), 3rd (63 colonies), and 4th (95 colonies) round of bio-panning. The periplasmic extract from these 190 cultures, induced with IPTG to express Nbs (fused with the HA-tag and His-tag at their C-terminus) was tested in a PE (periplasmic extract) ELISA on wells coated with or without rPasB. The positive clones were analyzed by colony PCR and sequencing. For screening of clones from the synthetic library, 285 individual colonies were selected from the 2nd (95 colonies), 3rd (95 colonies), and 4th (95 colonies) round of bio-panning. PE-ELISA was performed against antigen protein. The positive clones were analyzed by colony PCR and sequencing. Finally, seven different Nbs (sNb42, sNb43, sNb44, sNb410, sNb31, and sNb462 from the synthetic library and Nb747 from the immune library) have been identified (Figure S3).

### 2.3. Expression and Purification of Selected Nbs

**Figure 2:**
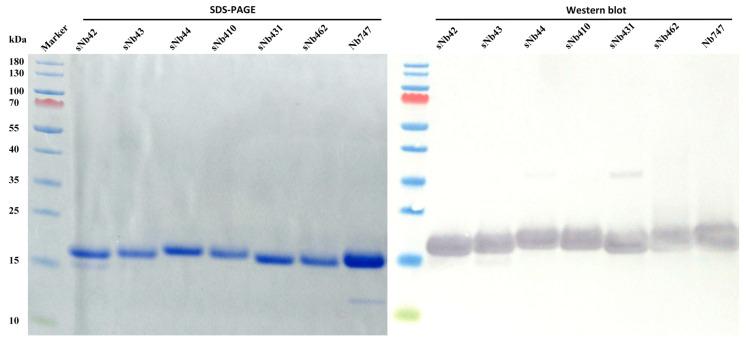
Confirmation of the specific Nbs. SDS-PAGE and Western blot of the His- and HA-tagged Nbs after expression and purification by IMAC and SEC.

The genes of the selected Nbs from the synthetic library were subcloned into pMECS vector for expression and purification. The plasmids containing the genes of the Nbs were transformed into WK6 cells to produce the Nbs with a HA-tag and a His-tag at their C-terminus. Soluble Nbs were extracted from the periplasm by osmotic shock and purified by IMAC and SEC. Yield of selected Nbs was indicated in Supplementary Materials Table S1. As shown in Figure 2, Coomassie staining after SDS-PAGE revealed single bands with molecular mass of around 15 kDa as expected from their amino acid sequence and demonstrated high purity and integrity of Nbs. The presence of the His-tag in these Nbs was confirmed by Western blot after visualizing the single bands.

### 2.4. Characteristics of Nbs

#### 2.4.1. Thermal Stability

The thermal stability of the Nbs was assessed by a thermofluor assay. As summarized in Table S1 and Figure S4, the thermal stability indicated Tm values ranging from 57.5 (sNb42) to 72.2 °C (sNb410), which reflected the high thermo stability of the retrieved Nbs. The sNb44 and Nb747 showed a high stability with a Tm value of 66.4 °C and 68.1 °C, respectively.

#### 2.4.2. Affinity to Antigen

The apparent affinity of the selected Nbs against their target was estimated via saturation ELISA binding studies against immobilized antigen in the wells. The Nbs were incubated with coated antigen at different concentration. The Nb concentration when absorbance signal dropped to 50% of the maximum value corresponded to the apparent affinity of the Nb- antigen interaction. As shown in Table S1 and Figure S5, Nbs selected from synthetic library (sNb43, sNb44, sNb410, and sNb462) and Nb747 possess an affinity in the single digit nM range for their cognate antigen.

#### 2.4.3. Specificity of Nbs against Antigen

**Figure 3:**
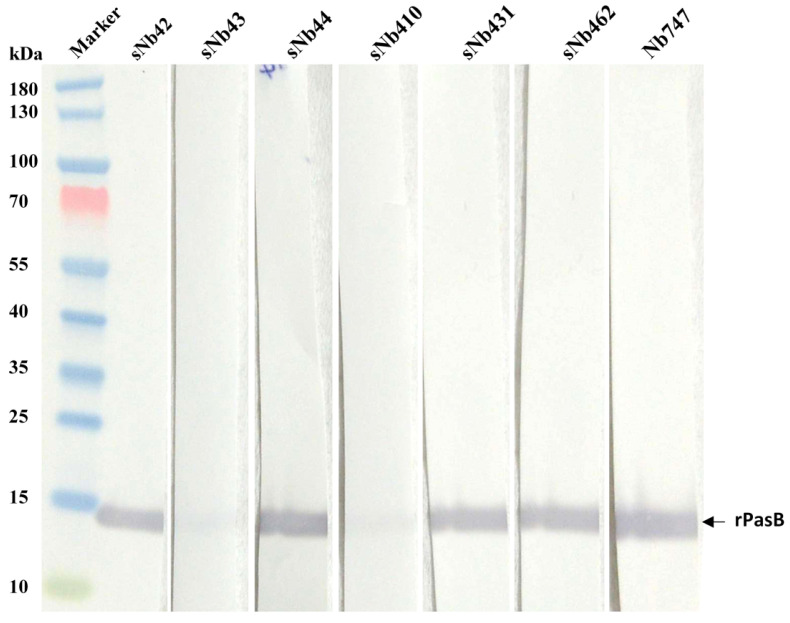
Antigen-specificity of the Nbs. Purified rPasB protein was loaded on the SDS-PAGE and transferred to a nitrocellulose membrane. The membrane was cut into different strips and incubated with the Nbs. The presence of the Nb was detected with monoclonal anti-HA IgG and HRP conjugated anti-mouse IgG.

The specificity of selected Nbs was verified by Western blot after SDS-PAGE under reducing conditions. The result demonstrated the targeting of the selected Nbs to the band corresponding to the expected size for rPasB, which indicated the potential recognition of these Nbs to rPasB (Figure 3).

### 2.5. Stabilization of Endogenous HIF-1α

**Figure 4:**
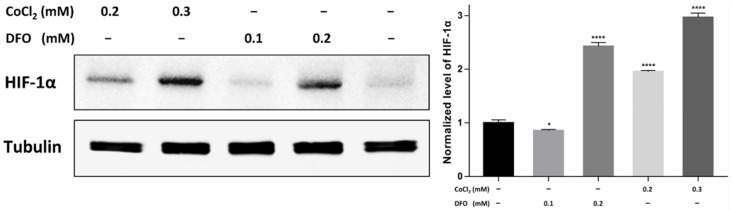
Determination of the condition to stabilize endogenous HIF-1α protein in HeLa cells via Western blot. HeLa cells were stimulated with the condition indicated on the figure or left untreated. Then, cells were collected and lysed by freeze-thaw cycles. The released cytoplasm was separated by SDS-PAGE and transferred to a nitrocellulose membrane for Western blot analysis. Mouse anti-HIF-1α IgG and anti-tubulin IgG were incubated with the membranes and HRP conjugated anti-mouse IgG was used to confirm the presence of the bands. The statistical difference in the figures is indicated as follows: * (*p* < 0.05), **** (*p* < 0.0001).

The endogenous HIF-1α protein was first stabilized by stimulating HeLa cells with different concentration of desferrioxamine (DFO) and cobalt chloride (CoCl_2_). The optimal condition to protect HIF-1α from pVHL degradation in HeLa cells cultured in presence of various concentrations of DFO and CoCl_2_ was revealed after visualizing the cellular HIF-1α protein content by Western blot. The relative expression was reflected after normalization to the internal reference, i.e., tubulin. The result revealed the diverse efficiency to stabilize endogenous HIF-1α protein after stimulating with DFO and CoCl_2_ (Figure 4). It was observed that the highest level of HIF-1α was protected after treating HeLa cells with 0.3 mM CoCl_2_. In the group of untreated HeLa cells, the weak band represented the background level of endogenous HIF-1α before degradation by pVHL.

### 2.6. Interaction of Selected Nbs with Native HIF-1α

**Figure 5:**
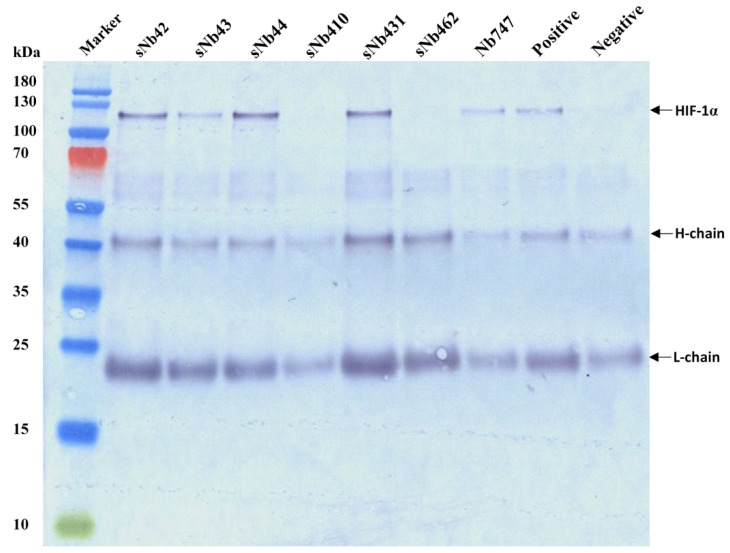
Immunoprecipitation of HIF-1α protein by selected Nbs. HeLa cells stimulated with CoCl_2_ were harvested and lysed with freeze-thaw cycles. The cell lysate was collected and incubated with Nbs to capture HIF-1α protein. Protein A/G coated agarose beads were incubated with monoclonal anti-His tag IgG to precipitate the Nbs from HeLa cell lysate. The precipitation was loaded on the SDS-PAGE and transferred onto a NC membrane for detection of the native HIF-1α via Western blot with mouse anti-HIF-1α IgG and HRP conjugated anti-mouse IgG. Immunoprecipitation with mouse anti-HIF-1α IgG instead of Nbs was performed and served as the positive control. Immunoprecipitation with mouse anti-His tag IgG instead of Nbs was performed and served as the negative control. The light and heavy chains from mouse anti-His IgG were indicated as ‘L-chain’ and ‘H-chain’, respectively.

To determine the interaction of Nbs with the native HIF-1α, an immunocapturing analysis was performed to check the targeting of Nbs to the endogenous HIF-1α protein in HeLa cells after stimulating with 0.3 mM CoCl_2_. After capturing, the protein complex was separated by SDS-PAGE and then transferred onto nitrocellulose membrane for Western blot analysis. The bands corresponding to HIF-1α were visualized after incubating with mouse anti-HIF-1α IgG and HRP conjugated goat anti-mouse IgG. As shown in Figure 5, some of the selected Nbs (sNb42, sNb43, sNb44, sNb431, and Nb747) could potentially capture HIF-1α protein, which demonstrated the recognition of these Nbs of the native HIF-1α.

### 2.7. Expression of Intrabodies and Inhibition on HIF-1 Related Target Genes

**Figure 6:**
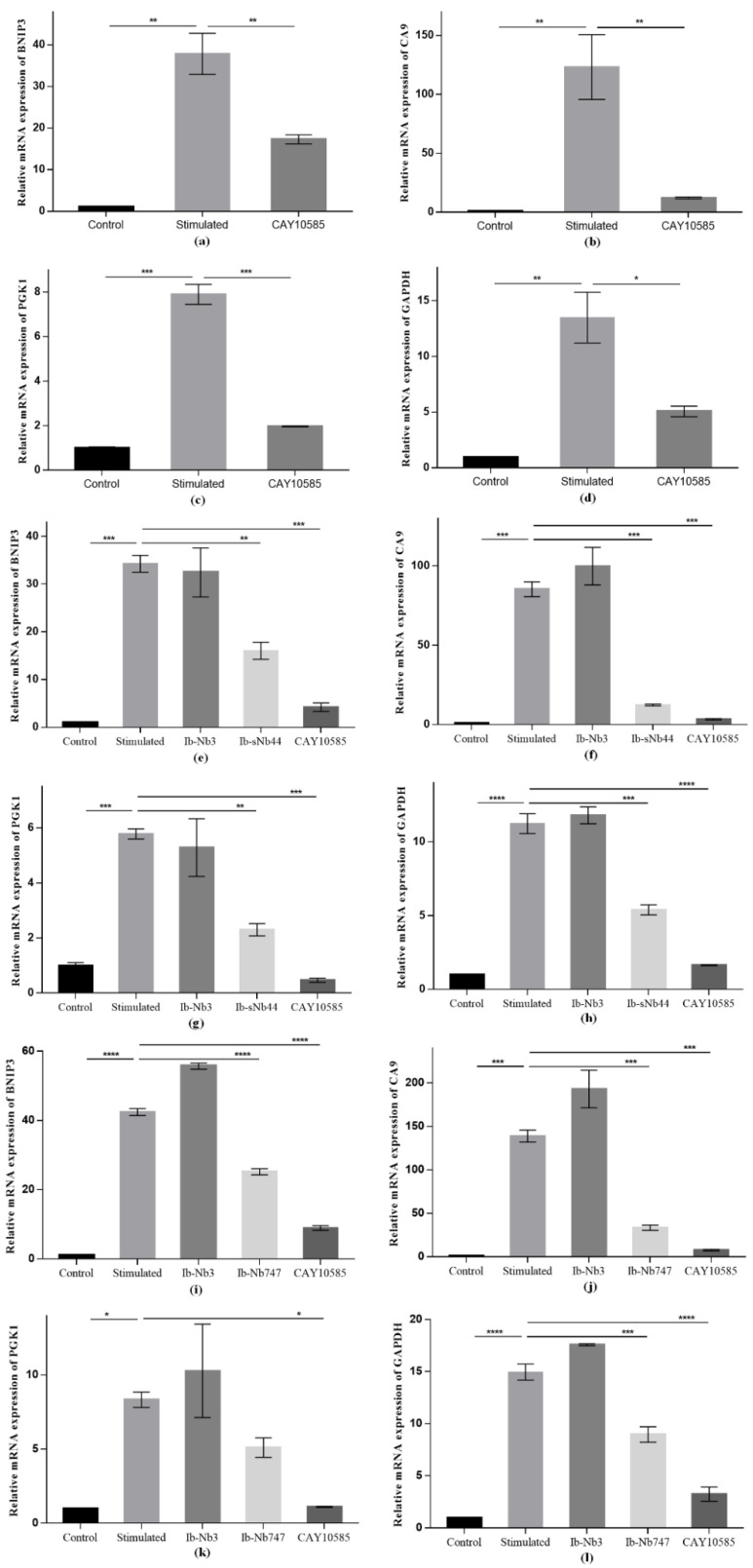
Expression level of HIF-1 related target genes. HeLa cells were stimulated with 0.3 mM CoCl_2_ for stabilization of endogenous HIF-1α protein. HIF-1 related target genes of *BCL2 interacting protein 3 (BNIP3)*, *carbonic anhydrase 9 (CA9)*, *phosphoglycerate kinase 1 (PGK1)*, and *glyceraldehyde 3-phosphate dehydrogenase (GAPDH)* were selected to evaluate the inhibition of the transcriptional activity of HIF-1. The chemical inhibitor CAY10585 was selected as the positive agent to stimulate the HeLa cells to inhibit the activity of stabilized HIF-1α protein. (**a**–**d**) The model of HeLa cells stimulated with CoCl_2_ was confirmed, and the gene expression level of *BNIP3* (**a**), *CA9* (**b**), *PGK1* (**c**), and *GAPDH* (**d**) was monitored relative to expression of beta-2-microglobulin (B2M). (**e**–**h**) HeLa cells with stable expressed intrabody of Ib-sNb44 was used to determine the inhibition efficiency after simulating with CoCl_2_. HeLa cells with stable expressed irrelevant intrabody Ib-IRNb3 was used as the negative control. The inhibition of Ib-sNb44 on target genes was confirmed, and the gene expression level of *BNIP3* (**e**), *CA9* (**f**), *PGK1* (**g**), and *GAPDH* (**h**) was indicated. **i**–**l**: HeLa cells with stable expressed intrabody of Ib-Nb747 were used to determine the inhibition efficiency after simulating with CoCl_2_. The inhibition of Ib-Nb747 on target genes was confirmed, and the gene expression level of *BNIP3* (**i**), *CA9* (**j**), *PGK1* (**k**), and *GAPDH* (**l**) was indicated. The statistical difference in the figures is indicated as follows: * (*p* < 0.05), ** (*p* < 0.01), *** (*p* < 0.001), and **** (*p* < 0.0001).

Nb747 and sNb44 were selected to prepare intrabody expression constructs. Nb3 recognizing a bacterial chaperon protein was used as negative control and verified without interaction with rPasB in previous study [35]. The intrabody expression constructs were then transfected into HeLa cells for intracellular expression of Nbs. The expression of intrabodies was verified by Western blot. As indicated in Figure S6, the bands corresponding to the size of around 15 kDa were observed after detecting their fused His-tag. The level of HIF-1α protein in HeLa cells with intrabody expression was determined by Western blot, and the result was indicated in Figure S7 as bars after analyzing the intensity of the blots. The results revealed that the increased level of HIF-1α protein after stimulating with 0.3 mM CoCl_2_, whereas no significant variation of HIF-1α protein level was observed for cells with intrabodies expressed or not, which revealed that no significant intervention on the level of HIF-1α protein was observed after expression of Nbs (sNb44, Nb 747, and Nb3) in HeLa cells. For the analysis of the inhibitory efficiency of intrabodies on HIF-1 related target genes, the gene expression level in the hypoxic model was verified to ensure the applicability of the HeLa cells after stimulating with CoCl_2_. Meanwhile, a positive control was set up to determine the variation of the target genes after inhibiting the activity of HIF-1α by CAY10585, which is a chemo-inhibitor of HIF-1α. As shown in Figure 6a–d, the expression level of target genes was dramatically increased after stimulating HeLa cells with 0.3 mM CoCl_2_, and the expression returned to background levels upon inhibiting HIF-1α activity with CAY10585, which demonstrated that the established model could be applied for the analysis of the inhibitory efficiency of intracellularly expressed Nbs, and CAY10585 could be used as the positive control. Then, the inhibition by intrabodies was assessed after performing quantitative real-time polymerase chain reaction (qRT-PCR). As indicated, the inhibition on the expression of HIF-1 related target genes could be observed for both Nbs of Ib-sNb44 (Figure 6e–h) and Ib-Nb747 (Figure 6i–l). The differential inhibitory efficiency for Ib-sNb44 and Ib-Nb747 on the various target genes of HIF-1α could be detected, which reflected the different activities of these two intrabodies on inhibition of HIF-1α. No significant variation of expression level of target genes was observed after transfecting the irrelevant Nb of Nb3 in HeLa cells, which verified the lower interference of the transfection on the transcription in HeLa cells.

## 3. Discussion

As a hallmark in solid tumors, hypoxia is related to the unsatisfactory therapeutic outcome and poor prognosis [3,11,37]. HIF-1 is a key factor to activate transcription of target genes related to tumor cell growth and proliferation. It is a heterodimer of an α and a β subunit, and is considered to be an important target for diagnosis and targeted cancer therapy. The transcriptional activity of HIF-1 depends on the interaction and dimerization of PAS domains of the α and β subunits [23]. It was demonstrated that the deletion of PAS domain (PAS A and PAS B domains), either one of the domains or both, can dramatically decrease the function of HIF-1, and even a few amino acid mutations within the β-sheet of PAS B domain are sufficient to affect the activity of HIF-1 [24]. We therefore hypothesized that a small antibody fragment targeting the PAS domain to interfere the dimerization process, might be a feasible strategy to inhibit the transcriptional activity of HIF-1.

In the present study, we chose Nbs as candidates to target the PAS B domain of HIF-1α, in order to inhibit the transcriptional activity of HIF-1. For the selection of Nbs against HIF-1α, an immune library and a universal synthetic library were constructed. The same approach was applied to retrieve clones against the rPasB from the immune and the synthetic library by bio-panning. However, the synthetic library is much more diverse (9.63 × 10^10^) in comparison with the immune library (3.3 × 10^8^). The surface area of the microtiter plates normally used for bio-panning of the immune library is probably too small to allow all amplified virions from the synthetic library to contact the coated antigen protein. The risk of depleting and losing potential binders, especially during the first round of bio-panning, is high. We therefore employed an immune-tube providing a larger surface area to coat the antigen and to facilitate the interaction between the Nb displayed on phages and the coated antigen protein. We retrieved 6 Nbs from the synthetic library and 1 from the immune library that recognize specifically the recombinant HIF-1α PAS B domain. However, the expression yield of the selected Nbs varied significantly, with low yields of Nbs from the synthetic library, and a high yield for Nb from the immune library. The association of these Nbs to native HIF-1α was confirmed by immunoprecipitation from cytoplasmic protein extracts from HeLa cells stimulated with 0.3 mM CoCl_2_. It was demonstrated that five Nbs recognize the endogenous HIF-1α protein in HeLa cells. Therefore, binding of these Nbs to HIF-1α may block the key transcriptional activity of HIF-1 by intervening with the dimerization process of two subunits.

For analysis of the inhibitory efficiency, the intracellular expressed Nbs were prepared after transfection into HeLa cells [38]. The results confirmed the successful selection of the cells with Nb expressed intracellularly. Then, a hypoxia model to evaluate the inhibitory efficacy of the intrabodies to HIF-1 was established. Stimulating HeLa cells with 0.3 mM CoCl_2_ could stabilize the endogenous HIF-1α protein significantly. As reported, CoCl_2_ has the potential to occupy the iron center of the HIF-related proline hydroxylase, so as to inactivate the enzyme. In addition to this mechanism, CoCl_2_ could also bind to the sub-fraction of hydroxylated HIF-1α, thereby blocking the interaction between pVHL and hydroxylated HIF-1α, which prevents subsequent ubiquitination and eventually results in the stabilization of HIF-1α [39]. To assess the inhibitory capacity of our intrabodies on the transcriptional activation by HIF-1, several target genes (*BNIP3*, *CA9*, *PGK1*, and *GAPDH*) were selected based on the functionalities. Apart from being regulated by HIF-1, the proteins of these genes are also involved in tumor cell growth and proliferation. Therefore, these genes are established indicators to quantify the transcriptional activity of HIF-1 by qRT-PCR [34]. As mentioned before, the stimulation of HeLa cells with 0.3 mM CoCl_2_ stabilizes endogenous HIF-1α protein, and this raises the level of the target genes to adapt the tumor cells to the hypoxic environment, whereas CAY10585 is a well-known inhibitor of the activity of HIF-1α that decreases the level of the target genes.

The level of mRNA of our target genes in transfected HeLa cell after CoCl_2_ stimulation decreased in comparison with non-transfected cells. It was observed that Ib-sNb44 showed a higher inhibitory efficacy than Ib-Nb747, despite the higher thermostability and the higher affinity of the latter Nb. The targeted epitope of Ib-Nb44 might be more critical for optimal assembly of HIF-1 than that of Ib-Nb747 and therefore the former intrabody is a stronger inhibitor of the transcriptional activity of HIF-1. In the current study, as cells were incubated under normal oxygen tension, transfected HeLa cells with specific or irrelevant Nbs are not expected to exert any difference on the growth of cells. This was indeed confirmed by the culturing cells constitutively expressing Ib-sNb44, Ib-Nb747, or Ib-Nb3 for several months without apparent detrimental consequences. In order to evaluate the inhibition of HIF-1α specific Nbs to the growth of tumor cells, further analysis could be conducted to incubate under a hypoxia working station to visualize the growing state, and the inhibition efficacy of Nbs under such hypoxia conditions. Furthermore, a feasible delivery strategy still needs to be identified and developed to transfer Nbs into cells for the in vivo evaluation. Hereby, several Nbs were selected against HIF-1α protein and proved to be very promising to interfere the transcriptional activity of HIF-1. It may be worthy to explore the therapeutic potential of these Nbs in the future.

## 4. Materials and Methods

### 4.1. Strains, Cell Lines and Plasmids

*E. coli* TG1 strain was used for the construction of VHH libraries and production of phages. *E. coli* BL21(DE3) was used for recombinant protein production. *E. coli* WK6 was used for the periplasmic expression of selected Nbs. Phagemids pHEN4 [40] and pMECS [41] were used for the construction of a synthetic library and an immune library, respectively. Plasmid pET-21b (lab collection) was used to produce recombinant protein. HeLa cells were used to establish the model with stabilized endogenous HIF-1α protein. Cell lines were cultured in RPMI medium (Gibco, Paisley, UK) supplemented with 10% Fetal Calf Serum (Gibco, Paisley, UK), 100 U/mL penicillin-streptomycin (Gibco, Paisley, UK) and 100 U/mL L-glutamine (Gibco, Paisley, UK). Cells were cultured in an incubator with a humidified atmosphere of 5% CO_2_. pCI-neo mammalian expression vector (lab collection) with the G-418 selective marker was used to prepare intrabody expression constructs.

### 4.2. Production of Recombinant PAS B Domain Protein

The sequence of PAS B domain extended with His-tag at the C-terminal was designed based on the HIF-1α gene fragment and synthesized by Life technologies^TM^ after codon optimization. After digestion with Nde I and Not I, the fragment was ligated into pET-21b plasmid digested with the same restriction endonucleases, and then transformed into *E. coli* BL21(DE3) competent cells for rPasB protein expression. After overnight induction with 1 mM of IPTG (Duchefa Biochemie B.V., Haarlem, The Netherlands), cells were pelleted and lysed by sonication to release the cytoplasmic proteins containing rPasB. Recombinant protein was purified by IMAC on HisPurTM Ni-NTA Resin (Thermo Scientific, Waltham, MA, USA), and then further separated and desalted by SEC on a HiLoadTM 16/600 SuperdexTM 75 pg prepacked column (GE Healthcare, Chicago, IL, USA) equilibrated with Tris-Buffered Saline (TBS). Concentration of the purified protein was determined by UV-spectroscopy using Nanodrop. The purity of rPasB protein was determined by Coomassie stained SDS-PAGE, and Western blot was performed to confirm the presence of the His-tag.

### 4.3. Construction of Nb Libraries

Two libraries including an immune library and a synthetic library were constructed [42,43,44]. For the construction of the immune library, a young dromedary was immunized with rPasB protein to raise an immune response. The immunization process was performed by six subcutaneous injections at weekly interval, each with 100 µg purified antigen protein. Three days after the last injection, 100 mL anti-coagulated blood was collected from the jugular vein. Lymphocytes were isolated from this anti-coagulated blood on Leucoprep^TM^ tubes (Greiner Bio-One, Kremsmünster, Austria). The immune library was constructed according to the optimized protocol described previously [35]. Briefly, total RNA was extracted from lymphocytes and used as template in a reverse transcription polymerase chain reaction (RT-PCR) for cDNA preparation with oligo-dT_12–18_ primer (Thermo Scientific, Waltham, MA, USA). Then, a multi-step PCR was performed to amplify the VHH encoding gene fragments. In the first PCR, primers CALL001 (5′-GTC CTG GCTGCT CTTCTA CAA GG-3′) and CALL002 (5′-GGT ACG TGCTGT TGA ACT GTTCC-3′) are used and the amplicons separated on 1% agarose gel. The shorter amplicons were extracted from the agarose gel with QIAquick Gel Extraction Kit (QIAGEN, Hilden, Germany) and used as the template for the next PCR. The second PCR was performed with primers A6E (5′-GAT GTG CAG CTG CAG GAG TCT GGR GGA GG-3′) and PMCF (5′- CTA GTG CGG CCG CTG AGG AGA CGG TGA CCT GGG T-3′) with Pst I and Not I restriction enzyme. After purification and digestion with Pst I and Not I, the resulting PCR fragments were ligated into pMECS phagemid vector (digested with the same restriction enzymes) and transformed into *E. coli* TG1 electro-competent cells (Lucigen, Middleton, WI, USA) using the conditions recommended by the manufacturer (1800 V/cm pulse). After a recovery phase in ‘recovery medium’ (provided by Lucigen), TG1 cells were plated on LB agar petri dishes containing 2% glucose (m/v) and 100 μg/mL ampicillin for selection of transformants. After overnight incubation at 37 °C, colonies were scraped from the plates and cells were resuspended in LB medium supplemented with 20% glycerol (*v*/*v*) for storage at −80 °C. The percentage of cells with plasmids containing an insert corresponding to the size of a VHH was determined by colony PCR with primers MP57 (5′-TCA CAC AGG AAACAG CTA TGA C-3′) and GIII (5′-CCA CAG ACA GCC CTC ATAG-3′) on 95 randomly chosen colonies and visualization of the length of the amplicon by agarose gel electrophoresis.

For the construction of the synthetic library, the amino acid sequence was designed based on the scaffold of Nb BCII10, and the sequence of the CDRs was determined based on the sequence analysis of naturally occurring Nbs [36]. Bias was given to certain amino acids (i.e., Lys, Arg, Ser, Tyr, Asn, and Gly). The designed randomized gene was synthesized with the amino acid residues in CDRs represented as X in Figure S2. Each randomized position was mutated using a subset of trinucleotide blocks encoding: 10% for Arg, Lys, Ser, Asn, Gly, Tyr respectively, and a total percentage of 40% for all other residues in equal amounts. The random gene pool was synthesized and used as the template for PCR amplification. The steps similar as the construction of the immune library were applied for the synthetic library with the primer pair of LibF (5′-GTTCAGCTGCAGGAAAGCGGTGGTGGTAGCGTTC-3′) and LibR (5′-CGGGTAGCGGCCGCTCGAAACG-3′) (Pst I and Not I underlined). The gene fragments were then digested and subcloned into pHEN4 phagemid for the generation of the recombinant phagemids. After electroporation these phagemids into TG1 competent cells, the synthetic library was constructed.

### 4.4. Panning and Screening of Nbs

Specific Nbs against rPasB were retrieved from the immune library and the synthetic library after panning and screening processes [45]. Generally, one aliquot of the library stock was cultured in 300 mL 2 × TY medium supplemented with 1% (*w*/*v*) glucose and 100 μg/mL ampicillin until an OD_600nm_ of 0.6–0.8 was reached. Then, 1 × 10^12^ VCSM13 helper phages were used to infect the cells for 30 min at room temperature (RT) without shaking to allow phages to enter TG1 cells via their F-pilus. The infected TG1 cells were collected by centrifugation and the cell pellet was resuspended in fresh 2 × TY medium supplemented with 100 μg/mL ampicillin and 70 μg/mL kanamycin. After overnight shaking at 37 °C, phage particles in the culture supernatant were precipitated with polyethylene glycol (PEG)/NaCl and resuspended in a total volume of 1 mL sterile Phosphate Buffered Saline (PBS). The concentration of the resulting phage particles was calculated from the value of optical density at 260 nm (an OD_260nm_ of 1 = 3 × 10^10^ pfu/mL).

For enrichment of Nbs by biopanning, rPasB protein (100 μL at 100 μg/mL) was coated in one well of a microtiter plate (another well contained only coating buffer was used as a blank control). Residual protein binding sites were blocked by 4% skimmed-milk proteins, protein free buffer (Thermo Scientific, Waltham, MA, USA), 0.1% casein, or SEA BLOCK blocking buffer (Thermo Scientific, Waltham, MA, USA) in the sequential rounds of biopanning, to minimize enrichment of unspecific Nbs. About 1 × 10^11^ phage particles were added to the wells and incubated for 1 h at RT. After removing of non-adsorbed phages by stringent washing steps with PBS containing 0.05% Tween-20 (PBST), rPasB-bound phage particles were eluted with 100 μL of TEA solution (100 mM triethylamine, pH 11.0) for 10 min at RT. The eluted particles were transferred to fresh tube and immediately neutralized with 100 μL of 1.0 M Tris-HCl (pH 7.4). Ten μL of the eluted phage solution were serially diluted and added to TG1 cells for infection. After 30 min incubation at RT, infected TG1 cells were plated on square agar plates with proper antibiotics for selection and incubated overnight at 37 °C. The enrichment during consecutive rounds of biopanning was determined by comparing the number of colonies from antigen-coated and un-coated wells. The remaining fraction of eluted phages from the antigen coated well was used to infect exponentially growing *E. coli* TG1 cells to amplify phage particles for next round of panning. The panning was performed for 4 consecutive rounds of selection.

For screening of specific binders against rPasB protein, individual colonies containing TG1 cells were randomly picked and grown in 100 μL of 2 × TY medium supplemented with glycerol in round bottom culture plates. After overnight incubation at 37 °C, TG1 cells were inoculated to deep-well plates for growing, after shaking at 37 °C for 4–5 h, the cells were induced with 1 mM IPTG for the production of Nbs with HA-tag and His-tag. The periplasm, containing Nbs was released by freeze-thaw cycles and used to recognize rPasB protein coated in the microtiter plates. Each periplasm extraction was added to the antigen coated well and another well without rPasB, as the negative control. The binding potential of the Nbs was monitored by comparing the optical density of the antigen coated well with the negative control. The detection of Nbs bounded to antigen was performed with mouse anti-HA antibody and AP conjugated goat anti-mouse antibody (ImTec Diagnostics NV, Antwerp, Belgium). The positive colonies (at least 2-fold higher compared with the negative control) were grown and used for phagemid preparation, from which the Nb insert was sequenced and analyzed.

### 4.5. Expression and Purification of Selected Nbs

As the synthetic library was constructed in our pHEN4 phagemid, the genes encoding the Nbs from the synthetic library were sub-cloned into pMECS vector with Pst I and BstE II restriction enzymes for the production and purification of His-tagged Nbs. The selected Nb clones in the pMECS vector were transformed into *E. coli* WK6 cells for expression of the Nb fused to a HA-tag and His-tag at their C-terminal end. Briefly, WK6 cells were cultured in shake flasks containing 330 mL TB medium supplemented with 0.1% (*w*/*v*) glucose, 100 μg/mL ampicillin, and 2 mM MgCl_2_. The exponentially growing phase of cells was followed via measuring OD_600nm_. When the OD_600nm_ reached a value between 0.6 and 0.9, 1 mM IPTG was added to induce the Nb expression. Cells were then incubated for about 16 h at 28 °C to produce Nbs in the periplasm. Cells were pelleted by centrifugation and Nbs, expressed in the periplasm, were released by an osmotic shock. The periplasmic extract was loaded on a column with Ni-NTA resins (Thermo Scientific, Waltham, MA, USA) for affinity adsorption of Nbs with His-tag. After washing the non-captured proteins, Nbs were eluted from the resin with 500 mM imidazole in PBS, the elution was applied to a Superdex^TM^ 75 10/300 GL gel filtration column (GE Healthcare, Chicago, IL, USA) for further purification and desalting. The purity and identity of the proteins were determined by Coomassie stained SDS-PAGE and Western blot with mouse anti-His IgG (MA1-21315, Thermo Scientific, Waltham, MA, USA) and HRP conjugated goat anti-mouse IgG (PA1-28555, Thermo Scientific, Waltham, MA, USA). The concentration of Nbs was determined from the UV absorption at 280 nm and the theoretical extinction coefficient of the Nb was calculated from its amino acid content. The purified Nbs were stored at a concentration of 1 mg/mL.

### 4.6. Thermal Stability of Nbs

Thermal stability of Nbs was assayed via thermofluor in CFX Connect™ Real-Time PCR Detection System (Bio-Rad, Hercules, CA, USA). Stored Nbs were concentrated to 2.5 mg/mL on Vivaspin^®^ 2 mL Concentrators (Sartorius, Göttingen, Germany). The reaction system was prepared with 15 μL of Nb and 5 μL of 200× diluted SYPRO^®^ Orange Protein Gel Stain (Thermo Scientific, Waltham, MA, USA), and 10 μL of sterile PBS was added to the system to reach a final volume of 30 μL. The reaction was prepared in triplicate for each Nb, and system with PBS and SYPRO^®^ Orange Protein Gel Stain were used as baseline signal. The program was set up to increase the temperature from 25 to 95 °C at a rate of 0.5 °C/min. The data collected after running were analyzed in OriginPro 8 software with non-linear fitting to determine the melting temperature (Tm) of Nbs.

### 4.7. Affinity of Nbs

The apparent affinity of Nbs against rPasB protein was determined via saturation ELISA by coating rPasB protein on the microtiter plate. The plates were coated overnight at 4 °C with 100 µL rPasB per well at a concentration of 2 µg/µL in TBS. An additional row of wells without antigen served as a negative control. After rinsing, residual protein capturing sites in the wells were blocked with 200 µL 3% skimmed milk in PBS for 1 h at RT. Then, Nbs were incubated at various concentrations (10,000, 1000, 500, 100, 50, 10, 1, 0.1, 0.01, 0.001 nM) in 100 µL sterile PBS for 1 h at RT. After washing with PBST, 100 μL of 2000-fold diluted mouse anti-HA IgG was added and incubated. Subsequently, 2000-fold diluted HRP conjugated anti-mouse IgG was incubated as the secondary antibody and TMB as the substrate. The apparent affinity of the Nbs to rPasB was determined as the concentration of Nb that gave half of the maximal signal.

### 4.8. Specificity of Selected Nbs

Western blot was performed to determine the binding of Nbs to antigen protein. In total, 2 μg of purified rPasB was loaded onto a NuPAGE protein gel by using a one-slot comb. After performing an SDS-PAGE, separated proteins were transferred to a nitrocellulose (NC) membrane (GE Healthcare, Chicago, IL, USA) for Western blot. Blots were blocked with 5% skimmed milk in PBS and then cut into strips to allow the incubation with different Nbs (1 μg/mL) in sterile PBS. Then, the membrane was washed with PBST and incubated with mouse anti-HA IgG and HRP-conjugated goat anti-mouse IgG, sequentially. The binding of Nbs to antigen was visualized after developing with mixed HRP-substrate staining solutions (solution A: 6 mL methanol with 18 mg Chloro-1-naphtol; solution B: 30 mL TPA solution (500 mL: 14.63 g NaCl, 1.4 g Trizma base, pH 7.5) with 19 µL H_2_O_2_).

### 4.9. Stabilization of Endogenous HIF-1α Protein in Hela Cells

The level of endogenous HIF-1α protein is regulated by an oxygen-dependent degradation system. Hence, HIF-1α can be stabilized by culturing tumor cell lines under hypoxic conditions or through inhibiting the ubiquitous mediated degradation. It has been reported that both DFO and CoCl_2_ (Sigma-Aldrich, Burlington, MA, USA) can stabilize HIF-1α, although they appear to stabilize the HIF-1α via different mechanisms. DFO is an iron chelator, and the addition of DFO will inhibit hydroxylation of HIF-1α by chelating the iron required for the activity of related hydroxylases, whereas, CoCl_2_ prevents VHL binding during either the translation or the binding step. Cobalt can occupy the iron center of hydroxylases to inactivate the enzyme. Even if a portion of HIF-1α becomes hydroxylated, cobalt can still bind directly to the hydroxylated proteins to prevent the interaction between HIF-1α and VHL, thereby preventing the degradation of HIF-1α [39,46]. In this study, experiments were performed to identify the better candidate to mimic and produce hypoxic conditions in the culture. HeLa cells were grown to near confluency in 10-cm cell culture peri dishes and stimulated for 12 h with 0.1 mM DFO, 0.2 mM DFO, 0.2 mM CoCl_2_, 0.3 mM CoCl_2_, or left untreated respectively. Then, HeLa cells were scraped from the plates, collected, and applied on gel for Western blot to determine the best condition to stabilize HIF-1α. For Western blot, HeLa cells were cracked by freeze-thaw cycles to release the cytoplasm loaded and separated by SDS-PAGE. Proteins were transferred from the gel to a NC membrane and residual protein binding sites were blocked overnight with 5% milk in PBS. The NC membrane was then cut to separate the blot of HIF-1α from tubulin to allow the incubation with mouse anti-HIF-1α IgG (MA1-16517, Thermo Scientific, Waltham, MA, USA) and anti-tubulin IgG (ab44928, Abcam, Cambridge, UK) respectively. Next, the membranes were washed with PBST and incubated with HRP conjugated goat anti-mouse IgG in MPBS. The blots were developed by using an ECL Western blot substrate (Thermo Scientific, Waltham, MA, USA), and visualized with chemiluminescence of a ChemiDoc MP imaging system (Bio-Rad, Hercules, CA, USA) after optimized exposure. Concentration represented by the gray intensity of the blots was analyzed by using Image J, and the level of HIF-1α was determined after normalization to tubulin.

### 4.10. Immunoprecipitation of HIF-1a with Nbs

HeLa cells grown to near confluence in 10 cm cell culture dishes were stimulated for 12 h with 0.3 mM CoCl_2_. The cells were scraped and collected in immunoprecipitation buffer (IPB): 40 mM Tris pH 8.0, 1% Triton, 10% Glycerol, 280 mM NaCl supplemented with protease inhibitor cocktail (1000× Leu pepsin at 1 mg/mL, AEBSF at 30 mg/mL, EDTA at 100 mM). Collected cells were cracked by freeze-thaw cycles and were subsequently pelleted at 14,000 rpm for 10 min at 4 °C. In tube A, 200 µL supernatant of lysates was incubated with 3 µg of Nbs. Simultaneously, 2 µg mouse anti-His-tag IgG was incubated with 10 µL protein A/G plus-agarose beads (Santa Cruz Biotech, Dallas, TX, USA) in 200 µL IPB at 4 °C turned head-over-head overnight in tube B. One group with pure lysate without Nb in tube A was used as control. In another group, Nb was absent in tube A, and mouse anti-HIF-1α IgG was used in tube B to serve as positive control. Antibody coated protein A/G beads were blocked with 1% BSA in IPB at 4 °C for 20 min and washed once with IPB buffer. After centrifugation of tube B, the cell lysates with or without Nbs were used to resuspend the antibody coated agarose beads, and incubated overnight at 4 °C. The next day, beads were washed 5 times with IPB and resolved in 40 µL protein loading dye. Protein samples were separated by SDS-PAGE and analyzed with mouse anti-HIF-1a IgG and HRP-conjugated goat anti-mouse IgG by Western blot.

### 4.11. Cell Culture and Transfection

To generate the intrabody expressing construct, pMECS vectors containing genes of Nb747 and sNb4 were used as templates for PCR amplification with primers pCIneoNbF 5’-CCGCTCGAGATGGCCCAGGTGCAGCTGCAGGAAA-3’ and pCIneoNbR 5’-GGCTCTAGAGGGTGATGGTGATGGTGGTGGGAAC-3’ containing *Xho*I and *Xba*I restriction enzyme sites. The resulting PCR fragments were cloned into eukaryotic expression vector pCI-neo to produce the intrabody construct Ib-Nb747 and Ib-sNb44. An irrelevant Nb against *E. coli* FKBP-type peptidyl-prolyl cis-trans isomerase (SlyD) was used as a control: Ib-IRNb3 [35]. The resulting plasmids were controlled by restriction enzyme analysis and subsequently confirmed by sequencing.

HeLa cells were cultured with RPMI medium supplemented with 10% Fetal Calf Serum, 100 U/mL penicillin-streptomycin, and 100 U/mL L-glutamine at 37 °C with 5% CO_2_. Cells were stably transfected with intrabody constructs via electroporation. The HeLa cell line was passaged one day before the transfection to promote good proliferation and cell physiology. Intrabody constructs were treated with ethanol to purify and remove most of the endotoxin. For the electroporation, HeLa cells were harvested and counted when 80–90% confluence was reached. Then, cells were pelleted to allow for 6×10^7^ cells per sample and resuspended in 0.4 mL RPMI medium, supplemented with 20% FCS and L-glutamine and transferred into an electroporation cuvette. Five µg of intrabody constructs were added into the cuvette and mixed with HeLa cells. The electroporator was set up at appropriate condition (Voltage = 260 V, Capacitance = 850 µF, Resistance = 720 Ohms) and discharged. Then, HeLa cells were transferred to 20 mL RPMI medium supplemented with 20% FCS, L-glutamine and G-418 at a final concentration of 400 µg/mL for selection of transfectants. Cells were cultured at 37 °C with 5% CO_2_ into ’96-well’ cell culturing plates with 200 µL/well. Cells were incubated under selective condition and observed every other day. If needed, the medium was changed every 3–5 days. When the survivors kept growing to 70% confluence in the 96-well plates, cells were harvested and resuspended into 6-well cell culturing plates under the same selective condition. When the cells grown to 80% confluence, cells were transferred into 10 cm petri dishes under selective condition and incubated as before. When 80–90% confluence was reached in the petri dishes, the cells were harvested for the detection of intrabodies by Western blot, or stimulated and served for subsequent research purposes. For the detection of intrabodies, HeLa cells transfected with intrabody constructs were collected and lysed via freeze-thaw cycles. The supernatant was collected after spinning down and applied to SDS-PAGE gel for proteins separation. After transferring the proteins to a NC membrane, Western blot was performed with mouse anti-His-tag IgG and HRP conjugated anti-mouse IgG. The expression of intrabodies was determined after developing with solutions described above. For analysis of HIF-1α protein in HeLa cells with transfected intrabody constructs (Ib-sNb44, Ib-Nb747, and Ib-Nb3), Western blot was performed according to the steps described above. HeLa cells without any treatment serve as the blank control. HeLa cells stimulated with 0.3 mM CoCl_2_ were used as the control to indicate the variation of HIF-1α protein in HeLa cells after transfection with intrabody constructs. Internal reference of β-actin was used for normalization after label with anti-β-actin IgG (MA1-91399, Thermo Scientific, Waltham, MA, USA) and goat anti-rabbit IgGs (MA1-31460, Thermo Scientific, Waltham, MA, USA).

### 4.12. Inhibition on HIF-1α Induced Target Genes

qRT-PCR was performed to evaluate the inhibition of intrabodies on HIF-1α via determining the level of target genes. Several target genes of HIF-1 were selected based on the varied bioactivities, *BNIP3* [47], *CA9* [48], *GAPDH* [49], and *PGK1* [50]. The relative expression of target genes was normalized to the internal reference of B2M after detecting with SYBR green (Bio-Rad, Hercules, CA, USA). The model used for qRT-PCR analysis of HIF-1α induced target genes was established with HeLa cells stimulated by 0.3 mM CoCl_2_. The level of target genes before or after stimulation was evaluated via qRT-PCR to check whether stabilization of HIF-1α can raise the level of selected genes. CAY10585 (Abcam, Cambridge, UK), which is a chemo-inhibitor of HIF-1α, was used as positive control to check whether the level of target gene expression can be decreased after treating with CAY10585. Primers were designed and synthesized as shown in Table S2. The quality of the primers for qRT-PCR was evaluated with a melting program, and data were analyzed after normalization to B2M.

To analyze the efficiency of inhibition by intrabodies, different test groups were set up. HeLa cells with intrabodies were stimulated with 0.3 mM CoCl_2_ to stabilize HIF-1α and to be used as test groups. HeLa cells with Ib-IRNb3 were stimulated with 0.3 mM CoCl_2_ and used as the negative control to determine the effect of transfection to the level of selected target genes. Non-transfected HeLa cells were stimulated or left untreated, and used as control to determine the base level of target genes before or after stimulation by CoCl_2_. Stimulated HeLa cells treated with CAY10585 were used as positive control. After 12 h stimulation and incubation, RNA from total cell lysates was prepared by homogenization with Trizol reagent (Thermo Scientific, Waltham, MA, USA). The concentration and integrity of the extracted RNA was determined by Nanodrop. Sequentially, cDNA was prepared from 5 μg extracted RNA using Reverse Transcriptase (Thermo Scientific, Waltham, MA, USA) and oligo-dT_12–18_ primers. Data were analyzed, and figures were prepared with the software of GraphPad prism 8 (GraphPad Software, San Diego, CA, USA).

### 4.13. Statistical Analysis

Statistical analyses were conducted by using the one-way ANOVA, with Bonferroni’s multiple comparison test. The statistical difference in the figures is indicated as follows: * (*p* < 0.05), ** (*p* < 0.01), *** (*p* < 0.001), and **** (*p* < 0.0001).

## 5. Conclusions

We generated specific Nbs against HIF-1α PAS B domain as novel tools to interrupt the interaction of the two subunits and to inhibit the transcriptional activation of tumor related targeted genes of HIF-1. Intrabody constructs were prepared and expressed in HeLa cells. The inhibitory efficiency of these intrabodies was confirmed by qRT-PCR after demonstrating a net decrease of the mRNA level of genes induced by HIF-1.

## Supplementary Materials

The following are available online at https://www.mdpi.com/article/10.3390/ijms222212335/s1.
