# Supplementary material for: Intrabody Targeting HIF-1α Mediates Transcriptional Downregulation of Target Genes Related to Solid Tumors
Source: Int J Mol Sci. 2021 Nov 15;22(22):12335. doi: 10.3390/ijms222212335 (PMC8625554; doi:10.3390/ijms222212335)
Supplement: Supplementary file 1 [file ijms-22-12335-s001.zip › ijms-1443079-supplementary.pdf]

Supporting Information

Intrabody targeting HIF-1α mediates transcriptional downregulation of target genes related to solid tumors

Yaozhong Hu[1, 2], Ema Romão[2], Cécile Vincke[2], Lea Brys[2], Yvon Elkrim[2], Marylène Vandevenne[3], Changxiao Liu[4, *], Serge Muyldermans[2, *]

1. Research Institute of Public Health, School of Medicine, Nankai University, Tianjin, China.
2. Laboratory of Cellular and Molecular Immunology, Vrije Universiteit Brussel, Pleinlaan 2, 1050 Brussels, Belgium.
3. Chargé de recherche FRS-FNRS, Macromolécules biologiques, Centre d'ingénierie des proteins, Université de Liège.
4. State Key Laboratory of Drug Delivery Technology and Pharmacokinetics, Tianjin Institute of Pharmaceutical Research, Tianjin, China.

* Corresponding Authors:
Serge Muyldermans
serge.muyldermans@vub.be
tel: +32 2 629 19 69
Changxiao Liu
liuchangxiao@163.com
tel: +86 22 8535 8948

Table S1. Properties of selected Nbs

Nbs	MW	pI	Yield [a]	Tm value [b]	Affinity [c]
	kDa	—	mg/L	°C	nM
**sNb42**	15.72	7.97	1.5	57.5 ± 0.11	134.6 ± 1.40
**sNb43**	15.69	7.22	2.2	61.2 ± 0.10	6.9 ± 1.28
**sNb44**	15.98	8.84	2.4	66.4 ± 0.32	8.2 ± 2.04
**sNb410**	15.77	7.21	1.4	72.2 ± 0.24	4.7 ±1.99
**sNb431**	15.75	6.70	1.6	69.1 ± 0.07	67.8 ± 1.75
**sNb462**	15.78	7.96	2.4	68.8 ± 0.13	1.4 ± 2.01
**Nb747**	15.40	7.97	12.8	68.1 ± 0.27	1.4 ± 1.52

[a]: Expression yield of purified Nbs (in mg per liter of culture medium).

[b]: Apparent affinity of selected Nbs were represented as mean ± SD, and the test was repeated for 3 times (n = 3).

[c]: Thermal stability of selected Nbs were represented as Tm value generated from 3 repeated assay, with triplicates for every assay, and the data was illustrated as mean ± SD (n = 9).

Table S2. Primes of HIF-1 target genes for qRT-PCR analysis

Gene	Name	Primer
**HIF-1 target genes**	PGK1 F	5'-TGGGAACAAGGTTAAAGCCGA-3',
	PGK1 R	5'-AAAACCCACCAGCCTTCTGT-3'
	BNIP3 F	5'-GCCATCGGATTGGGGATCTAT-3',
	BNIP3 R	5'-GCCACCCCAGGATCTAACAG-3';
	CA9 F	5'-GCCTTTGCCAGAGTTGACGA-3'
	CA9 R	5'-TCTGAGCCTTCCTCAGCGAT-3'
	GAPDH F	5'-TGCACCACCAACTGCTTAGC-3',
	GAPDH R	5'-GGCATGGACTGTGGTCATGAG-3'
**Reference gene**	B2M F	5'-TGCTGTCTCCATGTTTGATGTATCT-3'
	B2M R	5'-TCTCTGCTCCCCACCTCTAAGT-3'

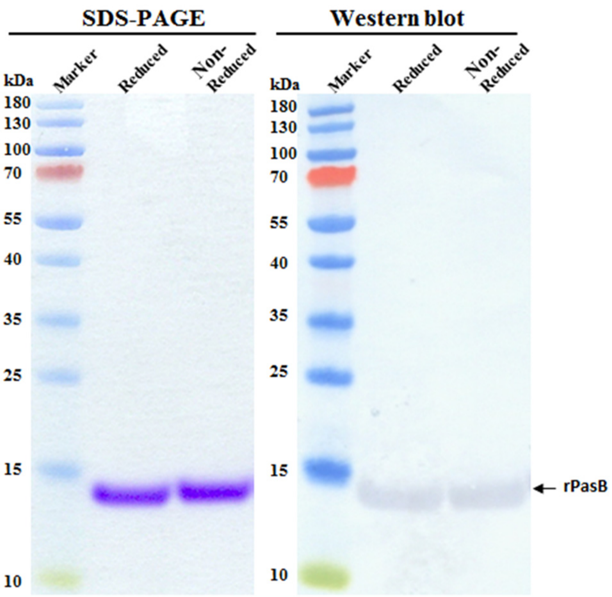

Figure S1. Identification of rPasB by SDS-PAGE and western blot. rPasB protein was loaded on gel and separated under reducing and non-reducing conditions. The bands were visualized by Coomassie staining and the presence of the His-tag was confirmed by western blot with anti-His tag IgG and HRP conjugated goat anti-mouse IgG.

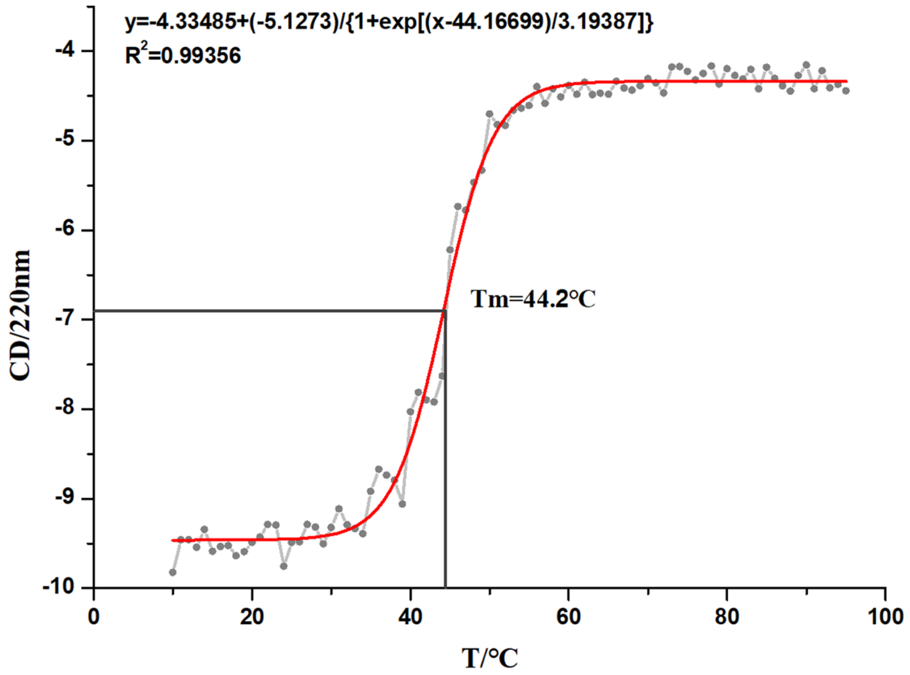

Figure S2. Thermal stability of rPasB. Temperature-dependent denaturation on the basis of changes in circular dichroism. The curve was analyzed via non-linear fitting, and temperature of 50% transition was 44.2°C for rPasB.

```
         1         10        20         30         40        50         60         70        80        90       100       110               120
IMGT     |********|*********|****** ***|******** *|*********|***** ****|***** ****|*********|*********|*********|**** *****|*12344321****** **|********
         <---------FR1-IMGT-------> <CDR1-IMGT-> <---FR2-IMGT----> <CDR2IMGT> <---------------FR3-IMGT-------------> <-----CDR3-IMGT-----> <FR4-IMGT->
sNb42    QVQLQESGG-GSVQAGGSLRLSCTAS GYWY----VPFS LGWFRQAPGQEREAVAA IASM--GGLT YYADSVK-GRFTISRDNAKNTVTLQMNNLKPEDTAIYYC AAFFGIKKL---NPSSSSVWY WGQGTQVTVSS
sNb43    QVQLQESGG-GSVQAGGSLRLSCTAS GYNY----MPFS LGWFRQAPGQEREAVAA IASM--GGLT YYADSVK-GRFTISRDNAKNTVTLQMNNLKPEDTAIYYC AAAFFGHLN---PPRSXVVWY WGQGTQVTVSS
sNb44    QVQLQESGG-GSVQAGGSLRLSCTAS GYRY----QYFS LGWFRQAPGQEREAVAA IASM--GGLT YYADSVK-GRFTISRDNAKNTVTLQMNNLKPEDTAIYYC AAFFGHKKL---PRSSVYYYY WGQGTQVTVSS
sNb410   QVQLQESGG-GSVQAGGSLRLSCTAS GYWY----YSFS LGWFRQAPGQEREAVAA IASM--GGLT YYADSVK-GRFTISRDNAKNTVTLQMNNLKPEDTAIYYC AAFFFGGHK---LNPPSSVYY WGQGTQVTVSS
sNb431   QVQLQESGG-GSVQAGGSLRLSCTAS GYWY----NLFS LGWFRQAPGQEREAVAA IASM--GGLT YYADSVK-GRFTISRDNAKNTVTLQMNNLKPEDTAIYYC AAAFFFGGH---LNPSSVYYY WGQGTQVTVSS
sNb462   QVQLQESGG-GSVQAGGSLRLSCTAS GYKY----WRFS LGWFRQAPGQEREAVAA IASM--GGLT YYADSVK-GRFTISRDNAKNTVTLQMNNLKPEDTAIYYC AAAAFFGGL---PQSSVYYYY WGQGTQVTVSS
Nb747    QVQLQESGG-GAVQAGGSLRLSCTAS GYTS----GTFS MGWFRQAPGKEREAVAG ISRG---GST YYPDSVK-GRFTISREVGKNTVYLLMNSLKPEDTAIYYC AAEEKLNPP--QRSSTTVWYY WGQGTQVTVSS
```

Figure S3. Amino acid sequence alignment of the Nbs. The amino acid sequences are aligned and numbered according to IMGT. The antigen binding loops or complementarity determining regions (CDR) and framework regions (FR) are indicated. The numbering of amino acids according to IMGT within the CDR3 is sequential from 105 to 117 for loops with length of 13 amino acids, with missing amino acids from 111 downstream and 112 upstream for CDR3 of shorter lengths and with insertions 111.1, 112.1, 111.2, 112.2 etc for longer loop lengths.

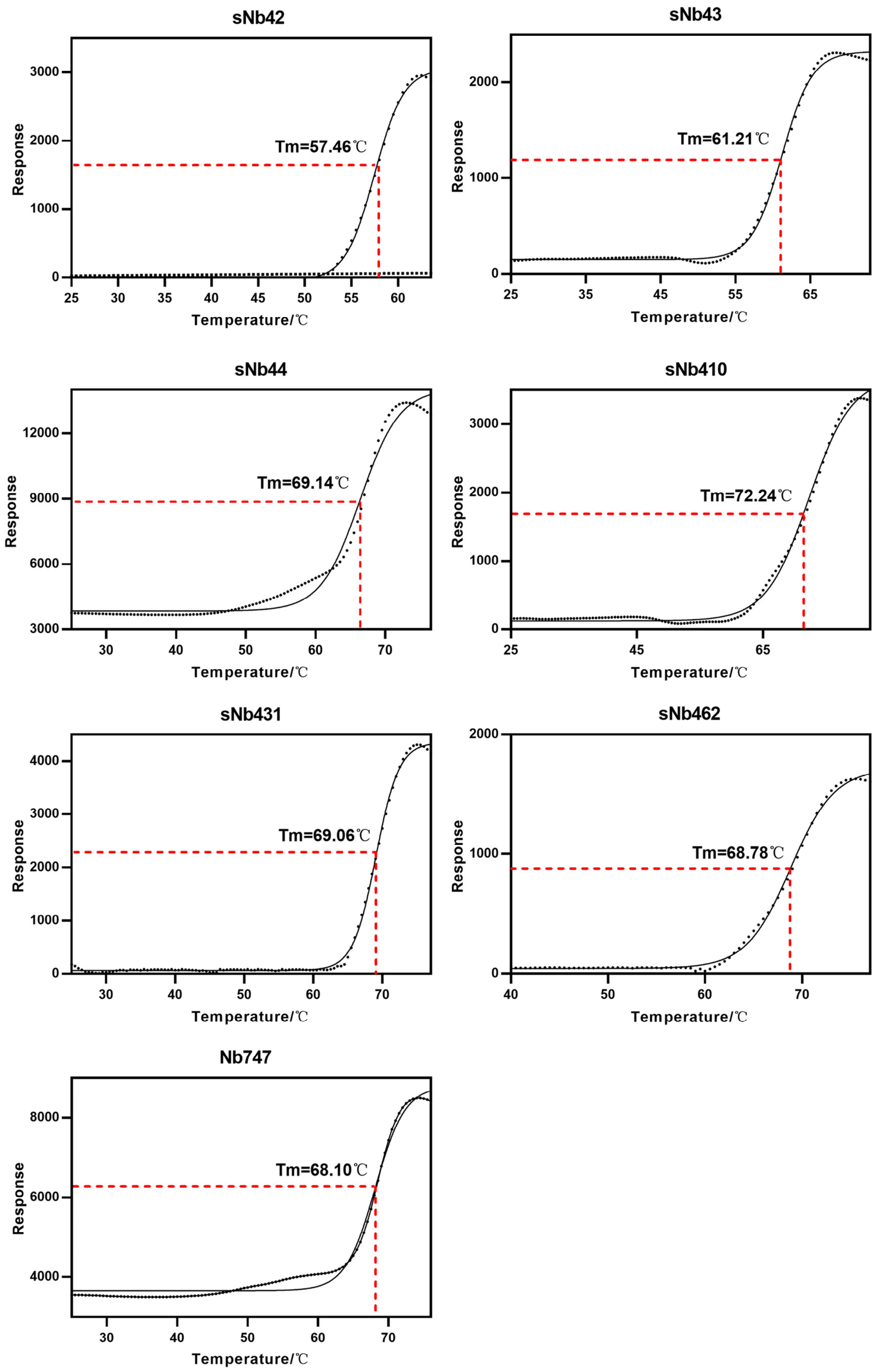

Figure S4. Thermal stability of selected Nbs. All data plotted are expressed as mean ± SD (n = 3). Repeated at least 3 times for every test.

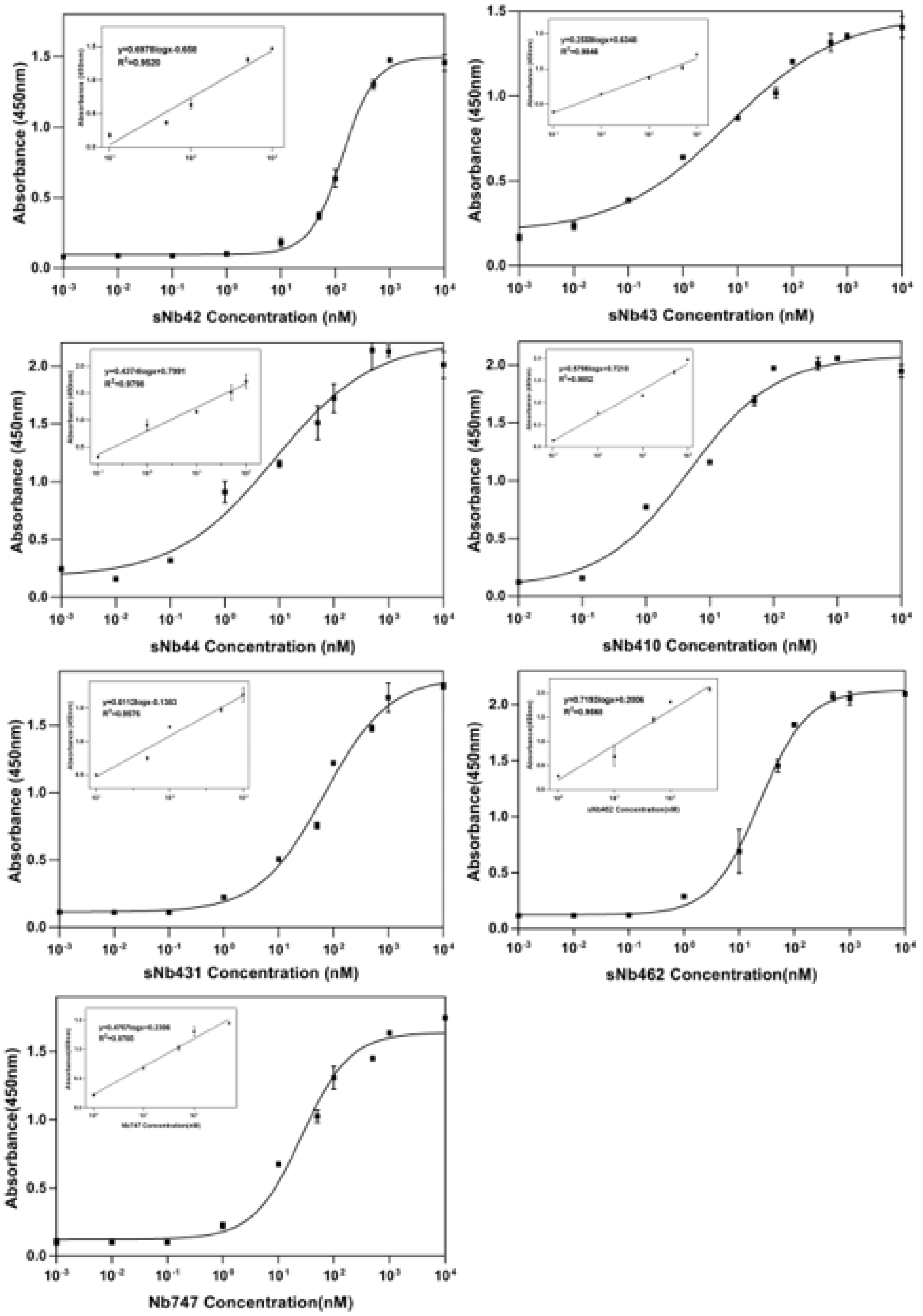

Figure S5. Binding affinity of selected Nbs. All data plotted are expressed as mean ± SD (n = 3). Repeated at least 3 times for every sample.

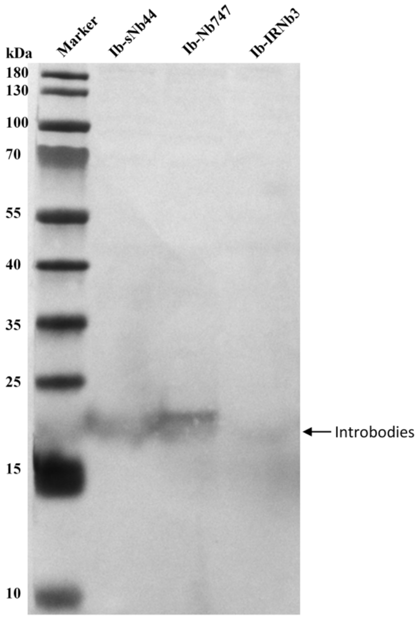

Figure S6. Detection of intrabodies expressed in HeLa cells by western blot. Intrabodies with His-tag were confirmed in HeLa cell lysate via western blot. A Nb targeting a HIF1-irrelevant antigen, referred to as Nb3, was intracellularly expressed as the negative control.

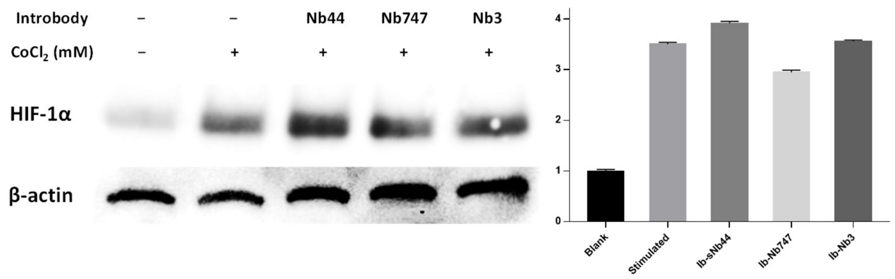

Figure S7. Level of HIF-1α protein in HeLa cells after expression of intrabodies. Western blot was performed to check the level of HIF-1α protein after expression of intrabodies of sNb44, Nb 747 and Nb3. HeLa cells without stimulating and intrabody expression served as the blank control. HeLa cells with stimulating of 0.3 mM $CoCl_2$ was used as the positive control to indicated the base level after stimulating. HeLa cells with intrabody expression were indicated as Ib-sNb44, Ib-Nb747 and Ib-Nb3.
